# Research and analysis of energy consumption and energy saving in buildings based on photovoltaic photothermal integration

**DOI:** 10.1038/s41598-024-51209-1

**Published:** 2024-01-09

**Authors:** Yahan Cui, Xinyan Zhang

**Affiliations:** 1 School of Architecture, The University of Sheffield, Sheffield, S10 2TN UK; 2 National Engineering Laboratory for Reducing Emissions From Coal Combustion, Engineering Research Center of Environmental Thermal Technology of Ministry of Education, School of Energy and Power Engineering, Shandong University, Jinan, 250061 China

**Keywords:** Environmental sciences, Energy science and technology, Engineering

## Abstract

In order to reduce the energy consumption of buildings, an air source heat pump assisted rooftop photovoltaic-thermal integration system is designed. The installation area of photovoltaic modules and collectors will not only affect the power side, but also affect the thermal side. Therefore, the basic architecture of the photovoltaic photothermal integration system is first established, and then the improved whale algorithm is used to optimize the photovoltaic photothermal integration system with the daily operating cost as the optimization goal. At the same time, the influence of the installation area of the photovoltaic photothermal module on the comprehensive performance of the system is analyzed, and the environmental and economic benefits of the photovoltaic photothermal system are analyzed. The results of the example show that the roof of the building has significant benefits in environmental protection and investment recovery period when the photovoltaic photothermal system with the optimal area ratio is installed on the roof of the building. The solar photovoltaic power generation system can reduce carbon dioxide emissions by 147.11 t within 25 years, and the solar collector system can save 170.5 thousand yuan in 1 year. It has achieved the purpose of saving energy, reducing carbon dioxide emissions and protecting the environment.

## Introduction

The energy crisis and environmental pollution are becoming more and more serious, and solar energy is getting attention because it is clean, non-polluting and widely distributed^1–3^. With the continuous improvement of photovoltaic power generation technology, photovoltaic solar-thermal integrated system has begun to be combined with building roofs^4^. The system does not take up additional space, and can be self-generated and self-consumed, and the surplus power can be fed into the Internet^5^. In the generation of electricity at the same time, can also use solar heating, near the user to provide hot water, energy-saving benefits are particularly obvious^6^. In high-rise buildings, the energy saving rate of building energy consumption is 16–58%^5,7,8^. Therefore, there is a great potential for energy saving in high-rise buildings. The value of energy consumption of people living in high-rise buildings is four times that of the average person in society. At the same time, some high-rise apartments have high density and long energy use time, which has become the main body of building energy consumption^9^. Combined with the characteristics of high-rise buildings, the introduction of roof photovoltaic photo-voltaic heat integration system into the energy-saving construction of high-rise buildings is of great significance in reducing energy consumption, promoting the application of green new energy and constructing green low-carbon buildings^10^.

Current research related to the utilization of solar energy mainly focuses on the integration with buildings. Alessandro et al.^11^ proposed to integrate solar photovoltaic photothermal integration with buildings organically. And use the external structure of the building to maximize the rational use of resources^12^. With the continuous development of photovoltaic photothermal technology, there are more and more forms of photovoltaic photothermal components combined with buildings^13^. Such as photovoltaic curtain walls, photovoltaic windows, photovoltaic roofs and so on. All of the above studies are combined with building materials that directly form part of the building^14^. However, for completed buildings, combining photovoltaic solar thermal modules with the building will destroy the existing structure of the original building and increase the investment cost. Therefore, for completed buildings, photovoltaic solar thermal modules can be installed separately on the roof. Power generation is realized on the basis of not changing the building structure and appearance^15^. In addition to photovoltaic solar thermal technology, solar collector technology in China has been more mature, solar thermal technology will be directly converted into heat energy^16^. However, the collector mainly depends on the amount of irradiation, in order to improve the collector in cloudy and rainy days, low irradiation limitations of heat production. Wei et al.^17^ proposed a new solar-assisted heat pump system, the solar heat pump unit and air source heat pump unit complement each other to run in tandem. It effectively solves the intermittency problem of traditional solar collector system and improves the utilization efficiency of solar energy and air energy. Hossein et al.^18^ proposed that the photovoltaic photothermal integration system can realize photovoltaic utilization and photothermal utilization at the same time, so as to improve the comprehensive utilization efficiency of solar energy. However, the system has high requirements for component materials and is difficult to maintain in the later stage. Shui^19^ optimized the photovoltaic power generation and solar hot water system for university buildings through the analysis of actual monitoring data. It is concluded that the carbon dioxide emission reduction in the system life cycle is about 3.8 kt^20^. However, the above system photovoltaic and photo-thermal systems are installed separately on different buildings on the campus, which cannot independently satisfy the electricity and hot water demand of a building.

On the basis of not changing the original building, the photovoltaic photo-voltaic heat integration system is now combined with the air source heat pump-assisted solar collector system. The photovoltaic photothermal integration system with solar energy as the main energy source is designed on the roof of the building. Simultaneously realizing the power supply and heating demand. An optimisation analysis of the installation ratio of the system based on the improved whale algorithm, where the installation area of the PV panels and collectors is used as an optimisation variable in order to maximise the economic efficiency of the system. Taking a high-rise building dormitory building as an example, a photovoltaic photo-voltaic heat integration system is installed on the roof to analyze the influence of the installation area ratio of photovoltaic photo-voltaic heat modules on the comprehensive performance of the system. As well as the economic and environmental benefits of the system, in order to provide a theoretical basis for building energy efficiency.

## Photovoltaic solar thermal integration system design

### System structure

The integrated photovoltaic-photothermal system consists of several parts, including a photovoltaic generator set, a collector and an air source heat pump. The input energy includes solar power generation, public grid electricity and collector heat collection. The operation principle of the system is mainly to generate electricity to meet the electrical load demand of the building through solar power generation equipment. The solar collector collects heat to realize the domestic hot water supply of the building^21^. The collector provides thermal energy (*Q*_*th*_), which is output to the system in the form of hot water. If the heat generated cannot meet the system demand, the air source heat pump is activated to supplement the supply (Q_a_). The photovoltaic power generation provides the system's electrical energy (*P*_*pv*_). When there is insufficient light intensity or weather conditions to meet the user's demand for power generation, the power is purchased from the grid (*P*_*buy*_), to make up for the difference in supply^22^. The user's electric load *P*_*load*_ and the energy consumption Pa of the air source heat pump are supplied by the PV and the grid. The thermal load Q_load_ of the user is supplied by the collector and the air source heat pump.

### System model

The photovoltaic solar thermal integrated system mainly uses solar energy as the main energy source, and the secondary energy source is the large power grid. The consumption of secondary energy is minimized as much as possible. The equipment of the system mainly consists of photovoltaic modules, collectors, and air source heat pumps. The mathematical model of each device is as follows^23^.

#### Solar irradiance at inclined surfaces

The amount of heat and power generated by the system is mainly determined by the amount of solar irradiation absorbed by the PV panels and collectors. The amount of solar irradiation at the tilted surface is mainly determined by Eqs. (1)–(4).

1 $$I_{T} = I_{{{\text{b}},T}} + I_{d,T} + I_{g,T}$$

2 $$I_{{{\text{b}},T}} = I_{{\text{b}}} \frac{{{\text{cos}}\theta }}{{{\text{cos}}\theta_{{\text{z}}} }}$$

3 $$I_{{{\text{g}},T}} = (I_{{\text{b}}} + I_{{\text{d}}} ){\text{p}}\left( {\frac{{1 - 2{\text{cos}}\beta }}{2}} \right)$$

4 $$I_{{{\text{d}},T}} = I_{{\text{d}}} (1 - F_{1} )\left[ {\frac{{1 - {\text{cos}}\beta }}{2}} \right] + I_{{\text{d}}} F_{1} \frac{{\text{a}}}{{\text{b}}} + I_{{\text{d}}} F_{2} {\text{sin}}\beta$$

where *I*_*T*_ is the solar irradiation on the tilted surface, *I*_b, T_, *I*_d, T_, *I*_g, T_ are the direct, scattered and reflected irradiation, *I*_*b*_ and *I*_*d*_ are the direct and scattered irradiation on the horizontal surface, *θ* and θ_z_ are the incidence angle and zenith angle of the sun, respectively^24^. *ρ* is the ground albedo, *β* is the tilt angle of the module, *F*_1_ and *F*_2_ are the number of the orbiting solar coefficients and the horizontal brightness coefficients, respectively. *a*, *b* are the correction coefficients of the solar incidence angle.

#### Photovoltaic power generation system

The power generated by the PV system is:

5 $$P_{{{\text{pv}}}} = \eta_{{{\text{pv}}}} I_{{{\text{pv}}}} S_{{{\text{pv}}}}$$

6 $$\eta_{{{\text{pv}}}} = 0.15\left[ {1 - 0.0045(T_{{{\text{pv}}}} - 25)} \right]$$

7 $$T_{{{\text{pv}}}} = T{\text{a}} + (T{\text{noct}} - 20)\frac{{I_{T} }}{800}$$

where: *S*_pv_ is the effective light-gathering area of the PV module. *η*_pv_ is the power generation efficiency. *I*_pv_ is the amount of solar irradiation obtained by the PV module. *T*_a_ is the ambient temperature. *T*_*NOCT*_ and *T*_pv_ are the nominal operating temperature and the operating temperature of the cell, respectively.

#### Vacuum tube heat collecting system

The heat collection capacity of the vacuum tube collector system is^25^:

8 $$Q_{{{\text{th}}}} = \eta_{{{\text{th}}}} I_{{{\text{th}}}} S_{{{\text{th}}}}$$

9 $$\eta_{{{\text{th}}}} = \frac{{A - B({\text{t}}_{{\text{w}}} - {\text{t}}_{{\text{a}}} )}}{I}$$

where: *S*_*th*_ is the effective light-gathering area of the collector. *η*_*th*_ is the collector efficiency. *i*_*th*_ is the amount of solar irradiation obtained by the collector. For a given type of collector, A and B are constants. *t*_w_ is the collector inlet water temperature. *t*_a_ is the ambient temperature. *i* is the unit solar irradiation.

#### Air source heat pump systems

The heat pump unit has a heating capacity of:

10 $$Q_{{{\text{ah}}}} = \frac{{{\text{gq}}_{{\text{r}}} {\text{p}}_{{\text{r}}} C({\text{t}}_{{\text{r}}} - {\text{t}}_{1} ){\text{k}}}}{T}$$

In Eq. (10): *Q*_ah_ is the hourly heating capacity. *g* is the daily water consumption. *k* is the safety factor. *T* is the daily working time. *q*_*r*_ is the design daily water consumption for hot water. *ρ*_*r*_ is the density of water. *t*_*r*_ is the temperature of hot water. *t*_*l*_ is the temperature of cold water. C is the specific heat of water.

## Methods of solving for different area ratios

### System control strategy

Based on the installation area of the PV panels and collectors^26^, the power generation and heat collection capacity of the system are calculated. When the heat generated by the collector cannot meet the demand of the building, the air source heat pump is switched on to supplement the supply^27^. When the PV power generation cannot meet the demand of the electric load, the power is purchased from the grid to supplement the supply difference, to achieve a balance between the supply and demand of the electric and heat loads. If the PV module and collector installation ratio is not reasonable system will produce a large amount of electricity and heat waste, resulting in economic losses. So, the main factor affecting the power generation and heat collection of the system is the installation area ratio of the modules. The solar photovoltaic photothermal system studied maximizes the use of solar energy resources with the help of photovoltaic and photothermal equipment under the premise of ensuring the safe operation of the system. For completed buildings, the available area of the roof is fixed, so the installation area of the modules is limited. To take into account, the principles of green and energy saving, it is necessary to rationally allocate the installation area of the modules.

### Solution methods

Optimisation algorithms are used in the solution process. The algorithms are transformed from the most basic particle swarm algorithms, genetic algorithms, etc. to newly developed algorithms. For example, ant colony algorithm, whale algorithm, etc. provide fast optimisation methods for finding the optimum for the objective. Two optimisation variables, Spv and Sth, are involved in the optimisation search process, and the whale optimisation algorithm has obvious advantages in terms of solution accuracy and convergence speed compared to meta-heuristic algorithms such as particle swarm and genetic algorithms^28^. And it has the advantages of fewer parameter settings and better optimisation seeking ability. However, it is unable to balance the local and global search ability, which will cause the loss of diversity in the late iteration, resulting in insufficient convergence ability. Therefore, the improved whale algorithm is adopted to optimise the calculation. When selecting individuals, the way of expanding the filter subset can be used to take the daily operating cost as the objective function. As much solar energy as possible is utilised for power generation and heat collection, and the size of power generation and heat collection is controlled by adjusting the installation area of PV modules and collectors. In order to meet the electrical and thermal load demand of the building, as little power as possible is purchased from the grid, so that the daily operating cost of the system is minimised.

### Algorithmic models

#### Objective function

Let *C*_pv_ and *C*_th_ be the selling price per unit of electricity and hot water respectively, then the formula for calculating the final return is:

11 $${\text{f}} = P_{{{\text{buy}}}} C_{{{\text{buy}}}} - \left( {\sum\limits_{{{\text{t}} = 1}}^{24} {P_{{\text{pv,t}}} C_{{{\text{pv}}}} + \sum\limits_{{{\text{t}} = 1}}^{24} {Q_{{{\text{th}},{\text{t}}}} C_{{{\text{th}}}} } } } \right)$$

where *C*_pv_ is the selling price per unit of electricity. *C*_th_ is the selling price per unit of hot water. *P*_*pv,t*_ is the amount of electricity produced by the photovoltaic system at time *t*. *Q*_*th,t*_ is the amount of heat produced by the collector system at time* t*. *P*_buy_ is the amount of electricity purchased, and *C*_*buy*_ is the price of utility electricity. Therefore, to maximise the economic benefits, as much solar energy as possible should be utilised.

#### Constraint condition

Power balance is the premise of stable operation of microgrid system, and its power constraint condition is:

12 $$\sum\limits_{{{\text{t}} = 1}}^{24} {(P_{{{\text{pv}},{\text{t}}}} + P_{{{\text{buy}},{\text{t}}}} )} = \sum\limits_{{{\text{t}} = 1}}^{24} {P_{{{\text{load}},{\text{t}}}} + \sum\limits_{{{\text{t}} = 1}}^{24} {P_{{{\text{ah}},{\text{t}}}} } }$$

In Eq. (12), *P*_*buy,t*_ is the amount of power purchased at the moment *t*. *P*_*load, t*_ is the power of the user load at the moment *t*. *P*_*ah, t*_ is the power consumed by the air source heat pump at the moment *t*.

13 $$\sum\limits_{{{\text{t}} = 1}}^{24} {(Q_{{{\text{th}},{\text{t}}}} + Q_{{{\text{sh}},{\text{t}}}} )} = \sum\limits_{{{\text{t}} = 1}}^{24} {Q_{{\text{load,t}}} }$$

where *Q*_*ah, t*_ is the heat generated by the air source heat pump at time *t*.* Q*_*load, t*_ is the heat required by the user at time* t*. The photovoltaic modules and collectors are mounted on the roof of the building and their area is constrained by the area of the building roof.

14 $$S_{{\text{pv - min}}} \le S_{{{\text{pv}}}} \le S{}_{{\text{pv - max}}}$$

15 $$S_{{\text{th - min}}} \le S_{{{\text{th}}}} \le S{}_{{\text{th - max}}}$$

16 $$S_{{\text{eff - min}}} \le S_{{{\text{th}}}} + S_{{{\text{pv}}}} \le S{}_{{\text{eff - max}}}$$

where *S*_*pv_min*_ is the actual minimum usable area of the PV module. *S*_*pv_max*_ is the actual maximum usable area of the PV module. *S*_*th_min*_ is the actual minimum usable area of the collector. *S*_*th_min*_ is the actual available minimum area of the collector. *S*_*th_max*_ is the actual available maximum area of the collector.* S*_*eff_min*_ is the actual available minimum area of the roof.

## Example analysis

### Calculation conditions

In order to verify the correctness of the proposed model and to find out the optimal setting of the system. A high-rise dormitory building is selected for the study, where the PV genset and solar collector are mounted on the roof of the building. There is no shading from tall buildings around the roof of this building and the roof surface is flat. Considering the shading problem, the actual usable effective area of the modules is 956 m^2^. Combining the geographic information and meteorological data of a high-rise building and analyzing and calculating with the help of the pvssyst software, the mounting tilt angle of the PV modules is set to be 36°, and that of the collectors is set to be 35.2. The solar irradiation is 5. 8 × 105J/(cm^2^ a). The heat transfer coefficient is 0.35–0.45 W/(m K). The shape factor is 0.7. Sunrise in Xinjiang is between 6 and 7 a.m. in the summer months, and sunset is between 9 and 10 p.m. The sunrise is between 6 and 7 a.m. in the summer months, and the sunset is between 9 and 10 p.m. in the summer months.

The power of the lamps in the public activity room, duty room, distribution room and other rooms is about 6 kw, and the power of the emergency equipment lighting and evacuation indicator light is 10 kw in total, so the load of the whole dormitory building is about 210 kw. Comprehensive home appliances are normally used at the same time and the use of coefficients, and the load of the whole dormitory building is 126–147 kw.

The building has 6 floors, and the interior of the dormitory is composed of 120 student dormitories (4 persons per dormitory), public activity rooms, duty rooms, public corridors, stairwells, and so on. The fixed hot water use time is 8:00–22:00, totaling 14 h. According to the hot water quota of the Design Code for Water Supply and Drainage in Buildings, the hourly heat consumption of the centralized hot water supply system is:

17 $$Q_{{{\text{load}}}} = \sum\limits_{{{\text{t}} = 1}}^{24} {{\text{q}}({\text{t}}_{{\text{r}}} - {\text{t}}_{1} )} {\text{p}}_{{\text{r}}} {\text{nb}}C$$

where *Q*_*load*_ for the hourly heat consumption. *q* for sanitary appliances hot water hourly water quota. *n* for the number of sanitary appliances, a total of 120.* b* for the same time the percentage of water, 70–100%.* t*_r_ for the hot water temperature, take the value of 55 °C; *t*_I_ for the cold-water temperature, take the value of 8 °C; *C* for the specific heat of water. The calculated hourly heating capacity of the system is 3471.36 MJ/h.

### Results at different area ratios

The solar photovoltaic solar thermal system is applied to the building and the optimisation results are obtained from the above data and equations. The main factors that constrain the power generation and heat collection of the system are the PV module and collector mounting area, and the building's electrical and thermal loads and the maximum mounting area will have an impact on the proportion of the modules to be installed.

**Figure 1:**
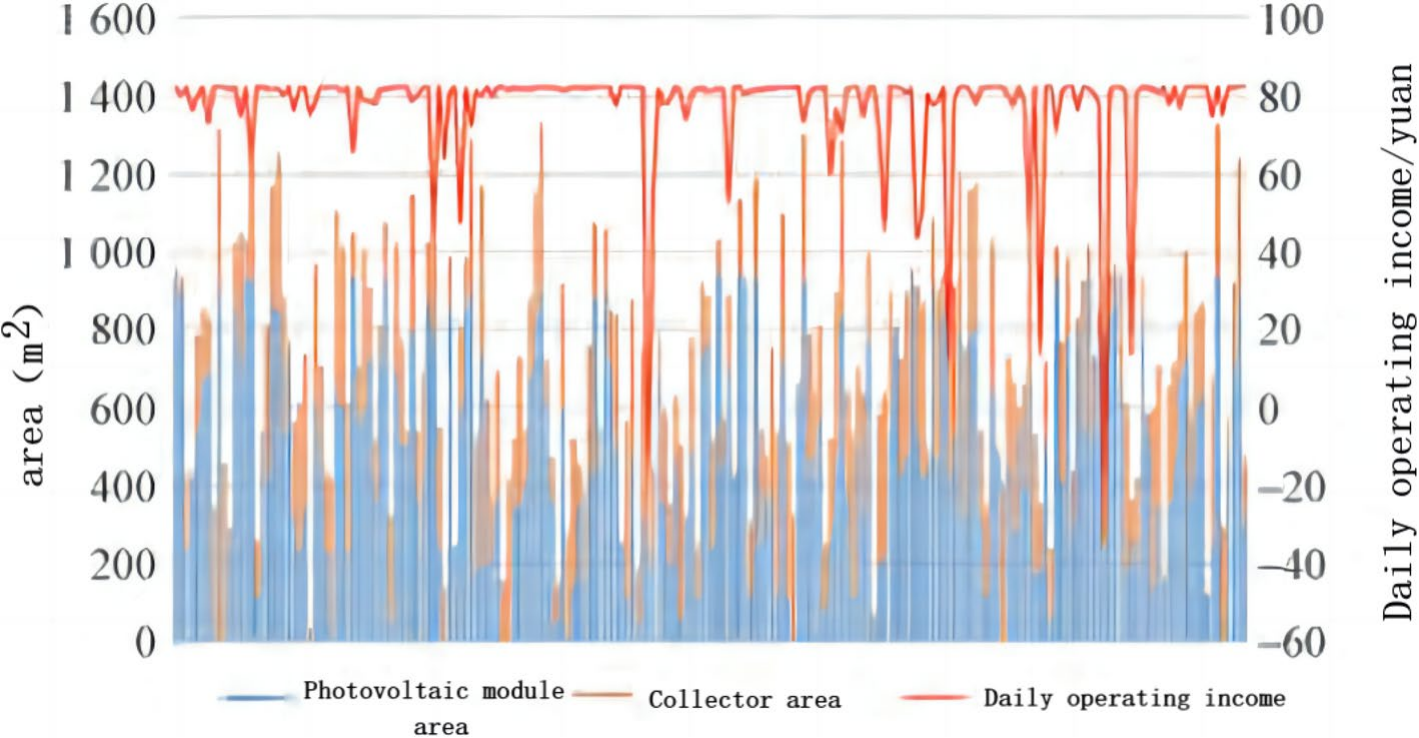
Daily operating revenues for different sizes of PV modules and collectors.

The analysis of the results of the calculation example reveals that the optimal installation area of the PV panels is 500 m^2^ with a daily power generation of 351.69 kW. The optimal installation area of the collector is 456 m^2^ with a daily heat collection of 3502.72 MJ. The daily operating benefits of the PV modules and collector with different installation areas are shown in Fig. 1. With the optimal ratio, the system can gain up to $82.44 per day of operation.

**Figure 2:**
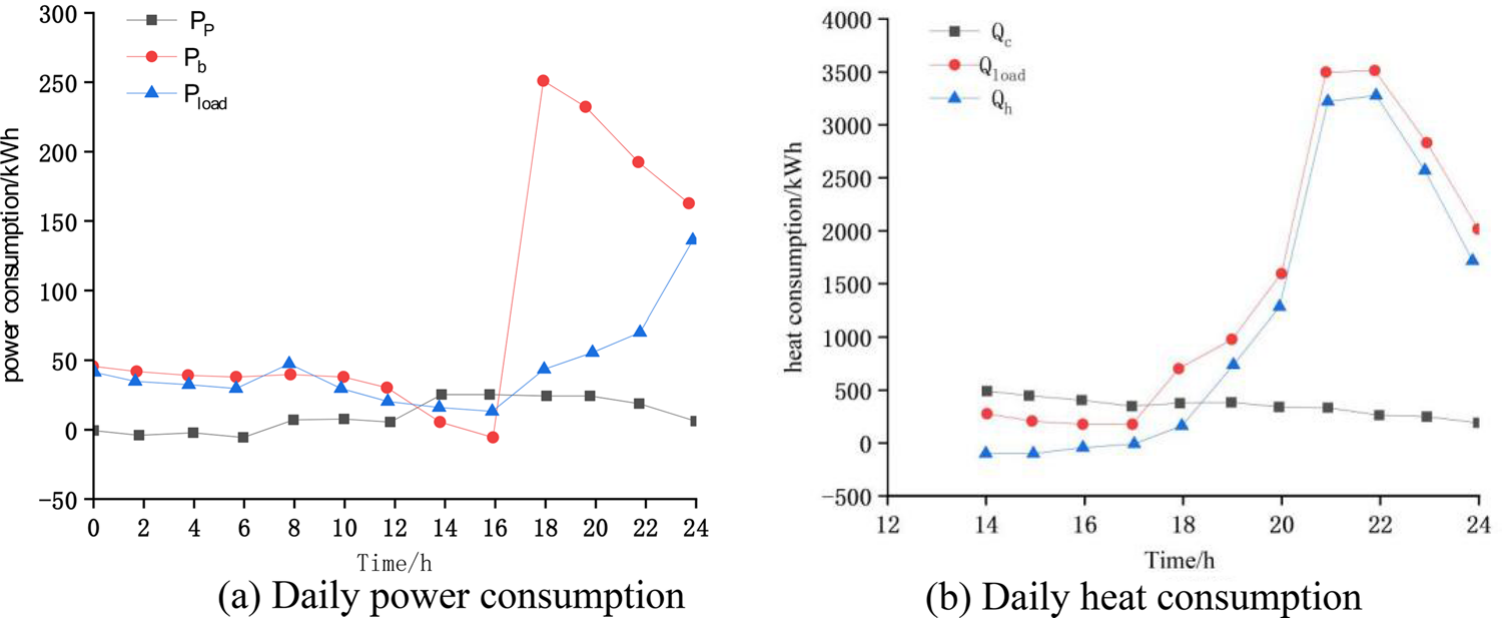
Variation curve of daily electricity and heat consumption.

The optimization process curves for both loads are shown in Fig. 2. From Fig. 2, the power generation of PV modules and the heat collection of collectors increase with the increase of solar irradiation. During the hours of 1:00am–7:00 am and 20:00 am–24:00 pm, there is no sunlight, so the light intensity is extremely low and cannot be relied on to provide electricity and heat from the sun, relying heavily on the grid and air source heat pumps. In addition, the amount of purchased electricity and heat appears negative, indicating that the energy provided by the system at this time is greater than the user's demand. However, the time of occurrence is short, the amount of electricity and heat is small and negligible. The sum of the heat provided by the collector and the heat provided by the air source heat pump is just equal to the user's heat consumption. The power provided by the photovoltaic and the power purchased from the grid is just enough to meet the user's load throughout the day as well as the power consumed by the heat pump to supplement the supply of hot water.

### Analysis of benefits

#### Benefit analysis of photovoltaic systems

The system cost consists of two main parts: system investment cost and system operation and maintenance cost, as shown in Table 1. The life cycle of the photovoltaic system is set to be 25a and the life cycle of the collector system is set to be 15a.

**Table 1:** System costs.

Photovoltaic system investment/($-m^−2^)	PV system operation and maintenance cost/$	Solar + air source Heat pump system investment cost/(yuan-m^−2^)	Solar + air source heat pump O&M costs/yuan
1305.64	Investment cost × 1 per cent	1482.56	Investment cost × 1 per cent

The average annual power generation capacity of the PV system is 128.4MWh, and the annual power generation capacity and income are calculated since the attenuation of the PV system will not be more than 20% during the whole life cycle of the PV system. At present, the national subsidy for self-generation and self-consumption in a high-level area, and the subsidy for residual power on-grid mode is RMB 0.42/kWh, and the income from self-consumption of electricity is RMB 0.9/kWh (the price of electricity consumption is calculated at RMB 0.48/kWh).

The economic benefits of the system 25a are shown in Table 2. According to Table 3, it can be concluded that the total power generation capacity of the solar PV power generation system in the whole life cycle is 2,834.5 MWh, the total revenue is 2,551,100 yuan, the total cost is 816,000 yuan, and the net benefit is calculated to be 1,735,100 yuan, and the cost of the system can be recovered in about 7.29 a. The system can be used to generate electricity for a period of 25 years.

**Table 2:** Economic benefits of photovoltaic power generation system 25a.

Time	Power generation/MWh	Income/ten thousand yuan	Time	Power generation/MWh	Income/ten thousand yuan
1	128.4	11.56	14	111.5	10.04
2	127.1	11.44	15	110.4	9.94
3	125.8	11.32	16	109.3	9.84
4	124.6	11.21	17	108.2	9.74
5	123.3	11.10	18	107.1	9.64
6	122.1	10.99	19	106.1	9.55
7	120.9	10.88	20	105.0	9.45
8	119.6	10.76	21	103.9	9.35
9	118.4	10.66	22	102.9	9.26
10	116.1	10.45	23	101.9	9.17
11	114.9	10.34	24	100.9	9.08
12	113.8	10.24	25	99.6	8.96
13	112.7	10.14	总计	2834.5	255.11

**Table 3:** Carbon emission factors.

Auxiliary energy	Coal	Oil	Natural gas	Electricity
F_0_	0.726	0.543	0.404	0.886

PV power generation process does not produce greenhouse gases and harmful gases, the environmental benefits are obvious. Each unit of electricity generated by the PV building is equivalent to 519g of carbon dioxide emission reduction, so the solar PV power generation system can reduce carbon dioxide emission by 1471.11 t in 25a.

#### Benefit analysis of solar water heating systems

Solar collector systems can save 1.3 × 109 kJ of energy a year, while 1 kW/h of electricity is converted into heat energy of 3600 kJ, and the price of electricity per kWh is 0.48 yuan. The solar collector system can save 170,500 yuan a year, and the total cost of the solar collector system is 777,400 yuan, saving 2,557,500 yuan in the whole life cycle. Solar water heating systems not only save conventional energy, but also reduce the emission of pollutants (mainly carbon dioxide).

18 $$Q_{CO2} = \frac{{\Delta Q_{{{\text{save}}}} {\text{n}}}}{{WE_{{{\text{ff}}}} }}F_{0} \frac{44}{{12}}$$

where *Q*_CO2_ is the carbon dioxide reduction over the full life cycle of the system. *W* is the standard heat medium value − 29.308 MJ/kg. *E*_*ff*_ is the efficiency of the conventional energy water heating unit. *n* is the system lifetime. *F*_*0*_ is the carbon emission factor as shown in Table 3.

The above system adds air source heat pump assistance to the solar water heating system. Therefore, the initial investment is larger than the conventional system, but the later operating cost is significantly lower than the simple solar water heating system. The auxiliary energy source for the hot water system in this project is electricity. From Eq. (18), the carbon dioxide emission reduction in the system life cycle is 2597.18 t, and the payback period of the collector system is 4.56 a. The system is designed to reduce the carbon dioxide emission in the life cycle of the system.

## Conclusions

In this paper, a rooftop solar photovoltaic (PV) photovoltaic integrated utilization system coupled with an air source heat pump is constructed. Based on the user's thermoelectric load characteristics, an optimization model is established with the daily operating cost as the optimization objective, and the installation area of photovoltaic modules and collectors is optimized. A high-rise dormitory building is taken as an example to build the proposed system, and the benefits are analyzed from the perspectives of environment and economy. The following conclusions are obtained.

1. In this paper, the improved whale algorithm is used to optimise the integrated photovoltaic solar thermal system. The PV power generation process does not produce greenhouse gases and harmful gases, and the environmental benefits are obvious. Each unit of electricity generated by the PV building is equivalent to 519g of carbon dioxide emission reduction, so the solar PV power generation system can reduce carbon dioxide emission by 1471.11t in 25 a.
2. On the basis of solar water heating system added air source heat pump auxiliary. Therefore, the initial investment is larger than the conventional system, but the later operating costs are significantly lower than the simple solar water heating system.

## Data Availability

All data generated or analysed during this study are included in this published article.
